# Radically different maxicircle classes within the same kinetoplast: an artefact or a novel feature of the kinetoplast genome?

**DOI:** 10.1186/1475-9292-5-5

**Published:** 2006-09-18

**Authors:** Pavel N Flegontov, Alexander A Kolesnikov

**Affiliations:** 1 Department of Molecular Biology, Lomonosov Moscow State University, Vorobjevy Gory 1, Build. 12, 119992 Moscow, Russia

## Abstract

We discuss here some results which suggest that radically different maxicircle classes coexist within the same kinetoplast. These data, although tentative and incomplete, may provide a new outlook on the kinetoplast genome structure and expression.

## Report

The kinetoplast genome is composed of a large heterogeneous population of minicircles and a small number of maxicircles. The maxicircles contain a conserved coding region of 16–17 kb and a non-coding "divergent" region which is highly variable in different strains and species [1-11]. It is believed that the population of maxicircles within a single mitochondrion is homogeneous.

The first results contradicting this widely accepted assumption were obtained by Lee et al. in the early 90-es [12-16]. Drastic changes of kinetoplast DNA were observed in strains of *Leishmania mexicana amazonensis *selected *in vitro *for arsenite or tunicamycin resistance, and these changes were accompanied by amplification of extrachromosomal DNA in the nucleus [12]. It was demonstrated that a new maxicircle DNA variant appeared in all drug-resistant strains along with the extrachromosomal DNA amplification. This variant was characterized by an altered pattern of restriction sites [12,13], it had a radically different sequence of the cytochrome *b *gene [13] and also displayed extensive DNA sequence rearrangements in the divergent region [16]. Nevertheless, the mutant cells demonstrated a normal growth pattern and apparently retained functioning mitochondria [13]. Moreover, alterations of the minicircle kinetoplast DNA component ("minicircle dominance switch") were also observed: the predominant wild-type minicircle class was replaced by other minicircle classes in the drug-resistant cells [12-15]. It was hypothesized that the switch of minicircle dominance might alter editing pathways and help to generate functional proteins from the altered maxicircle sequence (for example, of the cytochrome *b *gene) [13]. Remarkably, very similar maxicircle variants appeared in different cloned cell lines independently selected for arsenite or tunicamycin resistance [13,16]. This fact lead the authors to suppose that there was more than one maxicircle type in the kinetoplast DNA network in all studied cell lines, and a minor maxicircle variant became prevalent after the selection for drug resistance [13]. Thus, the switch of maxicircle dominance might accompany the switch of minicircle dominance. This conclusion remains controversial because, rather unexpectedly, minor maxicircle classes were not detected directly by PCR or hybridization in the wild-type or drug-resistant strains and because the results of Lee et al. were never repeated by another group. However, no alternative mechanisms underlying the observed drastic changes of the kinetoplast DNA were proposed.

The new results recently obtained by us [9] on a related species, *Leishmania major*, shed an additional light on the potential phenomenon encountered by Lee et al. with *L. m. amazonensis*. We observed that the promastigote-amastigote differentiation *in vivo *is accompanied by an alteration of the maxicircle divergent region sequence [9]. The DR structure proved to be drastically different at the promastigote and amastigote stages. Large-scale sequence rearrangements and also multiple small-scale insertions/deletions and single nucleotide substitutions were observed. Most remarkably, several strains of independent origin demonstrated an identical rearrangement pattern suggesting that the hypothetical "switch of maxicircle dominance" might take place during the differentiation. However, our results remain tentative, and this work needs to be repeated *in vitro *using clonal cell lines.

It is not known but possible that maxicircle genome activity differs between the amastigote and promastigote stages. We hypothesized that the structure of the divergent and/or the coding region in the prevailing maxicircle class may determine the pattern of genome activity. Thus, a novel regulatory mechanism may act in the kinetoplast of *Leishmania*: a specialized genome for each life cycle stage. This mechanism remains purely hypothetical, but because of its novelty and importance it deserves a thorough experimental verification.

The discussed results suggest that: 1) maxicircles may be intrinsically heterogeneous (at least in some *Leishmania *species); 2) different maxicircle classes may coexist within a single kinetoplast (since the strains of *L. m. amazonensis *used by Lee et al. were clonal); 3) maxicircle classes may have different divergent region structure and even different sequences of some genes; 4) a switch of the predominant maxicircle class accompanied by shifts in the abundance of minicircle classes might lead to alteration of editing patterns; 5) the maxicircle and minicircle dynamics might represent a novel mechanism regulating the kinetoplast gene expression. These hypotheses provide a new outlook on the kinetoplast genome expression and, therefore, deserve rigorous testing.

## Conclusion

We hypothesize that a switch of the predominant maxicircle class may take place during the promastigote-amastigote differentiation and under drug pressure. The data discussed here, although ambiguous and incomplete, suggest that a novel regulatory mechanism may operate in the kinetoplasts. Subsequent studies will either confirm the coexistence of radically different maxicircle classes within the same kinetoplast or provide an alternative explanation for the observed kinetoplast DNA alterations.

## Competing interests

The author(s) declare that they have no competing interests.
